# Recent Advances in the Use of Surface-Enhanced Raman Scattering for Illicit Drug Detection

**DOI:** 10.3390/s22103877

**Published:** 2022-05-20

**Authors:** Shamim Azimi, Aristides Docoslis

**Affiliations:** Department of Chemical Engineering, Queen’s University, Kingston, ON K7L 3N6, Canada; 19sa54@queensu.ca

**Keywords:** surface-enhanced Raman scattering, Raman spectroscopy, point-of-care diagnostics, drug detection, illicit drugs

## Abstract

The rapid increase in illicit drug use and its adverse health effects and socio-economic consequences have reached alarming proportions in recent years. Surface-enhanced Raman scattering (SERS) has emerged as a highly sensitive analytical tool for the detection of low dosages of drugs in liquid and solid samples. In the present article, we review the state-of-the-art use of SERS for chemical analysis of illicit drugs in aqueous and complex biological samples, including saliva, urine, and blood. We also include a review of the types of SERS substrates used for this purpose, pointing out recent advancements in substrate fabrication towards quantitative and qualitative detection of illicit drugs. Finally, we conclude by providing our perspective on the field of SERS-based drug detection, including presently faced challenges. Overall, our review provides evidence of the strong potential of SERS to establish itself as both a laboratory and in situ analytical method for fast and sensitive drug detection and identification.

## 1. Introduction

The increasing rate of illicit drug use and its consequences for the physical and mental health of individuals has become a major concern in recent years. According to the United Nations Office on Drugs and Crime (UNODC), around 275 million people used drugs in 2019, up by 22 percent from 2010, while almost half a million people lost their lives from drug use in that year [1]. In addition to fatalities, drug abuse can have various other irreversible physical and psychological consequences, including a weakened immune system, seizures, productivity losses, economic damage, and increased crime rates [2]. Rapid, accurate, and affordable drug detection and identification methods can be a powerful tool in our efforts to reduce devastating effects stemming from illicit drug use, or more generally, drug abuse. Drug detection methods can not only reduce fatalities among people who use drugs but also improve the safety and welfare of our society by assisting in the monitoring of trends and hotspots of drug abuse [3].

Numerous techniques have been employed for illicit drug detection in forensic toxicology, such as nuclear magnetic resonance [4,5], mass spectrometry [6,7], combined gas chromatography/mass spectrometry (GC-MS) [8,9], high-performance liquid chromatography (HPLC) [10], and X-ray powder diffraction [11]. In spite of their high molecular specificity, the aforementioned methods are typically centralized and require extensive sample preparation, expensive reagents, trained personnel, and time-consuming analysis. Such methods are not ideal for in situ drug testing or routine use by small publicly-funded organizations. Analytical techniques that combine user-friendliness with the ability for in situ sample testing have also been employed in illicit drug detection. Ultraviolet-visible (UV-Vis) spectrophotometry [3,12] is a simple and fast method; however, it provides a narrow spectral range and is only fit for the detection of a certain number of drugs [13]. Fourier transform infrared (FTIR) spectroscopy [14,15] suffers from water interference, while enzyme-linked immunosorbent assays [16,17] cannot match the low detection capabilities of the other methods. Commercial portable detection kits are currently available only for a limited number of drugs and require large sample volumes and specialized reactants [18,19].

Owing to their specificity and non-invasiveness, spectroscopic techniques, especially Raman spectroscopy, are highly effective in detecting trace amounts of illicit drugs in clinical and forensic applications. Raman spectroscopy is defined as a Category A technique, i.e., a technique with the highest amount of identification power for drug samples [20]. By contrast, ion-mobility spectrometry (IMS) is a Category B technique (less identification power), whereas color change tests are placed in category C (least). As a reagent-free, accurate, and rapid technique, Raman spectroscopy has been widely used for illicit drug detection in forensic analyses [21,22]. The presence of a conjugated ring system makes many drugs strong Raman scatterers, resulting in relatively large Raman cross-sections [23]. Databases containing the Raman “fingerprints” of many illicit drugs, such as cocaine [14,24] α-methyltryptamine hydrochloride, and 3,4-methylenedioxymethamphetamine (MDMA), also known as ecstasy [25], legal highs [26], and many of their analogues are already available. Owing to its small cross-section for water, Raman spectroscopy is not subject to solvent interference in aqueous solutions, which is a significant advantage over infrared spectroscopy for analyzing aqueous samples [27]. Raman spectroscopy’s sensitivity to the intrinsic molecular properties of analytes such as molecular structure, molecular weight, charge density, and functional groups allows the collection of quantitative and qualitative information for a given molecule [28].

However, the relatively low sensitivity of Raman spectroscopy is an impediment to its widespread use as a method for ultrasensitive detection and chemical analysis [29]. While typical cross-sections for absorption in the infrared and ultraviolet regions are ca. 10^−21^ and 10^−18^ cm^2^, respectively, per molecule, for Raman spectroscopy, the corresponding cross-sections are as low as ca. 10^−29^ cm^2^. This shortcoming can be overcome with the application of an alternative mode of Raman spectroscopy called surface-enhanced Raman scattering (SERS) [30,31] SERS is performed by placing the molecules to be detected in contact with a plasmonically active (metallic) nanostructure, often called the “SERS substrate” [32]. In SERS, when a molecule is adsorbed onto nano-roughened noble metal surfaces such as silver, gold, and copper, Raman spectra will be considerably amplified. Plasmonic nanoparticles (PNP), which are usually Ag, Au, or Cu, show strong surface plasmon resonance (SPR) in the visible to the infrared region and can generate a strong localized SPR (LSPR) effect. Hence, at the nanoparticle surface, the energy of the incident light is effectively coupled into the metal nanoparticles resulting in a considerable enhancement in the local electromagnetic field intensity, which is key to SERS intensity. The electromagnetic field enhancement preferentially appears in the sharp features, crevices, and gaps of the material’s surface. SERS can theoretically produce up to a 10^14^-fold signal enhancement, thus reaching single-molecule detection capabilities [33]. Unlike fluorescence, which exhibits broad adsorption/emission bands of the molecules, SERS has narrow spectral peaks. Moreover, the signal enhancement provided by SERS is necessary for the detection and identification of many drugs. For example, fentanyl and its analogues are often cut in less than 5.0 wt.% of the total sample, thereby becoming undetectable with conventional Raman. Moreover, the metabolite-to-parent drug concentration ratio helps determine the period the drug remained in the body, hence the time of last consumption. For the purpose of drug detection, SERS is a potential tool to instantly identify samples present in various forms, such as fiber textiles, nails, fingerprints, hairs, and beverages, which prevents the destruction of criminal evidence.

In the present article, we review the state-of-the-art use of SERS for chemical analysis of illicit drugs in aqueous and complex biological samples, including saliva, urine, and blood. We also include a review of the types of SERS substrates used for this purpose, pointing out recent advancements in substrate fabrication towards quantitative and qualitative detection of illicit drugs. Finally, we conclude by providing our perspective on the field of SERS-based drug detection, including presently faced challenges. Overall, our review provides evidence of the strong potential of SERS to establish itself as both, a laboratory and in situ, analytical method for fast and sensitive drug detection and identification.

## 2. Applications of SERS in Drug Detection

### 2.1. SERS Substrates for Drugs Detection Applications

Substrate choice is a critical factor in SERS. The intensity of the SERS signal depends highly on the geometrical features of the metallic nanostructure and its interaction with the molecule of interest [34]. Therefore, a great deal of effort has been invested into better understanding the effects of nanoscale features present on SERS-active substrates [35]. In particular, plasmon couplings in small gaps (1-10 nm) between plasmonic nanoparticles produce an intense electric field [36]. Ideally, a SERS substrate should produce a uniform and reproducible response, generate high enhancement, and be robust and straightforward to fabricate [14]. The SERS substrates that have so far been used for drug detection applications can be classified into two general categories: (i) colloidal systems and assemblies; (ii) and metallic nanostructures on flat, solid supports. 

#### 2.1.1. Colloidal Systems and Assemblies

The most commonly used SERS substrates are colloid-based due to their high stability, low cost, and ease of fabrication and implementation. Owing to the advances in nanotechnology and nanoscience, metallic nanoparticles with different shapes and sizes have been synthesized through chemical replacement [37], chemical reduction [38], thermal deposition [39,40], chemical deposition [41] and ultrasonic decomposition methods [42]. Due to their high surface plasmon resonance effect in the visible and the near-infrared wavelength ranges, colloidal silver and gold with sizes ranging from 10–150 nm are the most common metallic particles used for SERS-active substrates [29].

A typical chemical process for substrate fabrication is by reducing a metallic salt in the presence of sodium citrate [43]. However, other reducing agents such as hydrochloride, hydroxylamine, hydrazine, and sodium borohydride have also been used [44,45,46,47]. Generally, weaker reducing agents result in larger nanoparticles, while stronger reducing agents produce smaller nanoparticles. Colloidal substrates prepared by chemical reduction are commonly used to detect alkaloids such as cocaine, morphine, methamphetamine, and some structurally similar cannabinoids [47,48,49].

**Figure 1:**
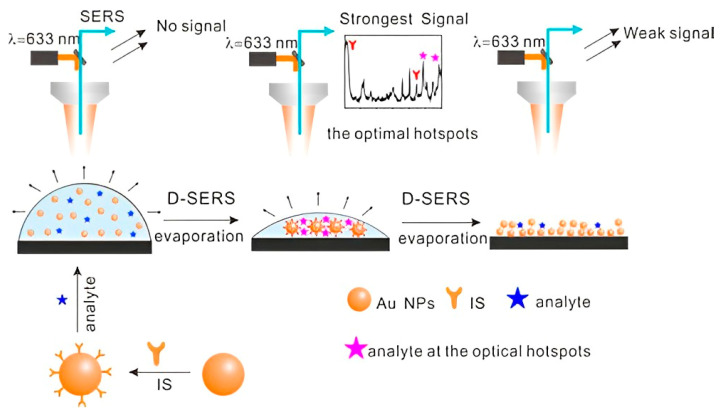
Schematic presentation of optimum hotspot formation during D-SERS, combined with the introduction of an internal standard for the detection of fentanyl. Sketches represent a drop of Au-sol, 3D hotspots, and aggregate of Au NPs, respectively [50].

Yan et al. prepared high-density hotspots with the optimum arrangement and excellent reproducibility through Dynamic Surface-enhanced Raman spectroscopy (D-SERS) (Figure 1) [50]. Dynamic Surface-enhanced Raman spectroscopy is based on two methods: solution-based and dry nanostructure film-based methods. The solution-based method involves transforming the nanoparticles from the wet state to the dry state, resulting in ordered three-dimensional (3D) hotspots, allowing for SERS detection with excellent reproducibility. Dry nanostructure film-based SERS detection is based on placing the colloidal nanoparticles on a solid substrate and sample drying on the substrate [51,52]. Using 4-mercaptopyridine as the internal standard, the resulting substrate exhibited great potential for the quantitative analysis of MDMA. Employing Ag colloidal particles produced by reducing silver nitrate hydroxylamine, Berg et al. showed differences in SERS and normal Raman spectra of amphetamine and amphetamine-H+ and between various conformers using ab initio model calculations [53]. Using silver hydroxylamine as the colloidal solution, Alharbi et al. detected tramadol in water and artificial urine with a limit of detection of 5.0 × 10^−4^ M and 2.5 × 10^−6^ M, respectively [54]. Carter et al. fabricated a SERS substrate from a colloidal silver solution to show the spectrum of freebase cocaine [24]. Haddad et al. reported the detection of fentanyl alone and as an adulterant in heroin using a paper-based substrate impregnated with silver nanoparticles [55]. Aggregation caused 1 to 2 nm gaps between silver nanoparticles, producing hotspots with 10^10^ enhancement factors. Burr et al. integrated Raman spectroscopy and paper spray ionization mass spectrometry (PSI-MS) with dual-purpose plasmonic paper substrates on a single-instrument platform [56]. Plasmonic papers were prepared by embedding paper swabs into the AgNPs colloidal solution. The modification resulted in a low detection limit of 0.6 ng and 1.0 ng for cocaine and fentanyl, respectively. The addition of Ag nanoparticles into polymer microgels gave rise to flexible SERS substrates, which can be further engineered into “smart” sensors with the incorporation of electrostatically charged or temperature-responsive molecules [57,58].

#### 2.1.2. Nanostructures on Flat, Solid Supports

Although metal nanoparticles are easy to produce, the fabrication of SERS substrates based on aggregated and dispersed metal nanoparticles for analytical applications is limited due to poor reproducibility and low enhancement factor. SERS substrates fabricated by immobilizing plasmonic nanostructures on planar surfaces provide a means of bringing nanoparticles in the vicinity of one another [59,60]. Planar substrates are suitable for integration in microfluidics and miniaturized devices. For some configurations of particles, such as spherical ones, aggregation is the critical factor in increasing the magnitude of the SERS effect [61]. In addition, type, size, orientation, interparticle distance in aggregated nanoparticles, roughness, and thickness of the film are critical factors for SERS performance [62,63]. In the following paragraphs, the assembly of metallic nanostructures on flat, solid supports is discussed.

Nanoparticle self-assembly. Self-assembly of nanoparticles over large areas (up to a few cm^2^) offers advantages such as high amounts of captured analyte. A variety of methods have been employed to self-assemble nanoparticles on solid surfaces. For example, the self-assembly of nanoparticles can be achieved through the chemical attachment of nanoparticles to a solid substrate. Zhang et al. achieved controlled self-assembly of gold nanoparticles into a 3D hotspot structure by regulating the addition of halogen ions [64]. Kang et al. implemented a bottom-up approach to achieve self-assembly of a close-packed gold octahedral array that exhibited an intense electric field at the edges and sharp corners of the nanostructure [65]. Ye et al. fabricated densely-packed, 2D arrays of plasmonic nanoparticles via nanoparticle self-assembly at the water-oil interface [66]. Small interparticle distances created highly stable, reproducible plasmonically active materials, enabling accurate quantitative SERS measurements.

Normally, bifunctional molecules are used to form a compact layer with the surface by one chemical moiety while having the other moiety interact with metal nanoparticles through chemical or electrostatic interactions. In this technique, capping agents play an essential role in preventing the aggregation of nanoparticles and forming a uniform layer. This method can also be employed to fabricate multi-layered nanoparticle structures [67]. After the deposition of the first layer, the surface is immersed in a ligand solution to act as a linker for the immobilization of subsequent layers [68,69]. The ligand molecules help control the interparticle distance to ∼1 nm and bring the nanoparticles close to one another [70]. However, the layers’ homogeneity cannot be totally controlled since the nanoparticles tend to cluster after the deposition of each extra layer [71].

**Figure 2:**
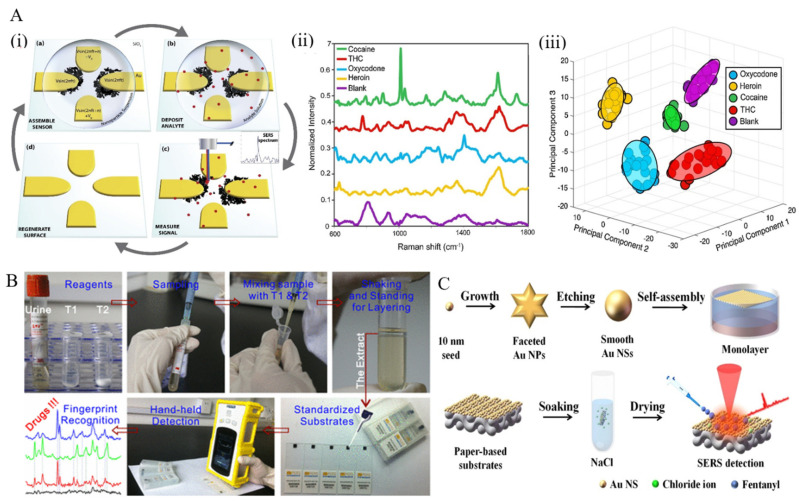
(**A**) (i) Schematic presentation of SERS-active substrates and detection process. (ii) Spectra obtained for illicit drugs tested and blank sample (water deposited on Ag dendrites). (iii) Plot of first three principal components used to cluster the spectra from different illicit drug analytes for identification. Each cluster consists of 20 spectra, and ellipsoids indicate a 95% confidence interval for each group [72]. (**B**) Illustration of a portable kit for rapid SERS detection of drugs in real human urine [74]. (**C**) Steps involved in the fabrication of gold nanoparticle cluster arrays using polystyrene-block-poly(2-vinylpyridine) (PSb-P2VP) templates on a silicon or glass surface [77].

Directed assembly of nanoparticles. Dies et al. presented a novel approach that provides ultrasensitive detection of illicit drugs [72]. Extended and interconnected dendritic nanostructures were fabricated through an electric field-guided assembly process of Ag nanoparticles, resulting in high hotspot density (Figure 2A). Additionally, the prepared substrate could act as a concentration amplification device by actively capturing analyte from the solution via electric field effects, thereby improving the detection of trace levels of illicit drugs. With the aid of statistical analysis methods (PCA and SVM), ultrasensitive identification and quantification were performed with almost 100% accuracy in detecting four different illicit drugs (heroin, THC, cocaine, and oxycodone). Dies et al. also employed electrokinetics to assemble nanoparticles on a scratched conductive surface [73]. Through the assembly of Ag nanoparticles on a scored conductive surface using an alternating current electric field, the formed substrate could detect trace levels of drugs, including cocaine and methanol, with high sensitivity.

In a novel work, Han et al. proposed a portable kit for in-field detection of amphetamine in human urine [74]. Highly reproducible two-dimensional (2D) Au nanorods were assembled by methoxymercaptopoly (ethylene glycol) (mPEG-SH) capping to enhance the adsorption of amphetamine to the gold surface, resulting in an excellent uniformity and reproducibility. The package consisted of a mini-Raman device and a platform for sample preparation to separate the analyte from human urine (Figure 2B). The detection limit for amphetamine was 0.1 ppm, and the device was employed for the accurate detection of MDMA and methcathinone in human urine, as well. 

Meng et al. also provided a way to quantitatively detect cocaine in human urine samples with self-assembled 2D gold nanoparticle films [75]. The nanoparticles were functionalized with CTAB, forming uniform close-packed nanoparticle films as a SERS substrate. High signal enhancement was achieved by producing sub-10 nm gaps for trapping the analyte. The device could detect cocaine with a limit of detection of 100 ppm and 500 ppm in an aqueous solution and a urine sample, respectively. Si et al. reported self-assembled soft and optically semi-transparent plasmene nanosheets to detect trace amounts of drugs [76]. Owing to their sharp edges, nanocubes were employed as the building blocks, offering additional SERS enhancement by means of the optical antenna effect. The resulted platform detected benzocaine with a detection limit of 0.9 × 10^−6^ M. 

Han et al. presented a paper-based SERS substrate decorated with uniform gold nanospheres for detecting fentanyl citrate in urine and serum [77]. The substrate was prepared by a liquid/liquid self-assembly technique, and chloride ions were applied to clean and modify the substrate surface. The resulting solid substrate had an enhancement factor and limit of detection of 1.64 × 10^5^ and 0.59 g/mL for the detection of fentanyl citrate in artificial urine, respectively (Figure 2C).

Electrostatic interactions are another means of assembling metal nanoparticles on solid supports [78]. This can be achieved by employing polymers as adhesives, e.g., polyelectrolytes [79], polyvinyl pyrrolidone (PVP) [80], polyvinyl pyridine [81], and polystyrene [82]. Biomolecules, such as DNA [83] and proteins [84], are used as linkers, sometimes acting as recognition elements. 

Yap et al. demonstrated the self-assembly of citrate-stabilized gold nanoparticles onto 2D, highly ordered arrays of uniform polyelectrolyte templates. Self-assembly was driven by electrostatic interactions between the negatively charged Au nanoparticles and the positively charged pyridinium groups on the silicon substrate prepared through the self-assembly of polystyrene-block-poly(2-vinyl pyridine) [85].

**Figure 3:**
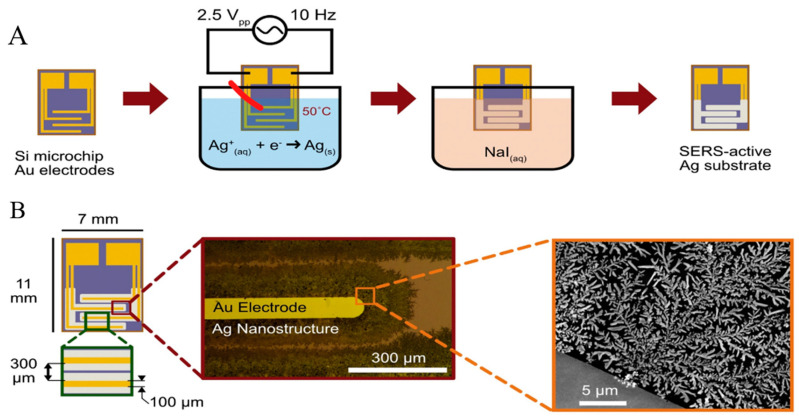
(**A**) SERS substrate assembly through reduction in Ag ions on the edges of interdigitated Au electrodes. (**B**) electrodes act as a template for the electrochemical growth of Ag nanostructures [88].

Electrochemical growth of nanostructures. Other methods, such as reducing ions on the substrate, have been employed to prepare solid SERS substrates [86,87]. Wilson et al. introduced a novel silver SERS substrate, assembled by electrochemically reducing silver ions onto a silicon chip with planar photolithographed gold electrodes, for the sensitive detection of fentanyl [88]. Through submerging Si-Au microchips into an aqueous solution of Ag^+^ and citrate ions and applying an AC potential to the microchip, SERS active silver nanostructures were formed onto the electrodes’ edge (Figure 3). The reported limit of detection for fentanyl was 0.078 ppm. 

### 2.2. Experimental Factors That Influence Substrate Performance 

The performance of a SERS system is subject to a number of parameters, which, if tuned properly, can enhance the plasmonic resonance of the metallic structure. Such parameters include the size, shape, surface modification, capping agent, type of solvent, and aggregation state of nanoparticles, all of which depend on metal salt, surfactant, reductant, pH, and reaction time [89]. However, the aforementioned factors can be interdependently addressed towards improving detection sensitivity (see also summary in Table 1).

Using a systematic approach, Mabbott et al. studied the effect of pH, aggregation time, and aggregation agent in SERS signal optimization to detect 5,6-methylenedioxy-2-aminoindane (MDAI) at very low concentrations [90]. The combination of KNO_3_ as the aggregation agent, pH 7.0, and aggregation time of 1800 s were found to produce the best SERS performance. Kline et al. also developed a colloidal SERS platform integrated into a microfluidic device to detect illicit drugs including methamphetamine, codeine, and morphine. The performance of the device was optimized by exploring the role of nanoparticle material, nanoparticle size, capping agents, and excitation wavelength on SERS signals and drugs’ detection limit [49].

Although colloidal assemblies can be physically adjusted to gain higher enhancement factors, the proximity of the analyte to the hot spot of the metallic structure also has a major effect on SERS detection ability. Enriching the analyte concentration in the proximity of SERS-active plasmonic surfaces through analyte manipulation or fabrication of hybrid materials can enhance SERS performance by 10–10^4^ fold [91]. Naqvi et al. fabricated a SERS substrate by reduced graphene oxide nanosheet decorated with silver nanoparticles (rGO/Ag NPs) through a simple wet chemical method [91]. The homogenous, stable substrate provided high-density hotspots for the SERS analyses.

Employing a novel approach, Liu et al. prepared a 3D hotspot matrix through evaporation of a droplet containing citrate and Ag ions on a silicon wafer to detect methylamphetamine (MAMP) and MDMA [51]. They concluded that their structure produced hotspots between every two nearby particles in 3D space and provided an excellent structural basis to trap analytes and molecules. In another work, a SERS-active substrate was prepared by assembling individual Au nanoparticles onto the surface of Ag nanowires using spontaneous capillary imbibition [92]. In addition to the ease of fabrication and the ability to form evenly spatially distributed hotspots, the Ag nanowires coupled with Au nanoparticles acted as an optical antenna. The single hotspot prepared a nanochannel to trap the molecules to the capillary imbibition, resulting in the high-sensitivity detection of cysteamine and adenosine-50-triphosphate down to 10.0 nM.

Surface functionalization is a practical approach for ensuring the selectivity of colloidally assembled SERS substrates. Employing molecules or chemical groups that can exclusively bind moieties (i.e., viruses, proteins, and antibodies) have been employed and resulted in more selective detection of target analytes in the presence of interferents that can overlap with the specific target signals [93].

**Figure 4:**
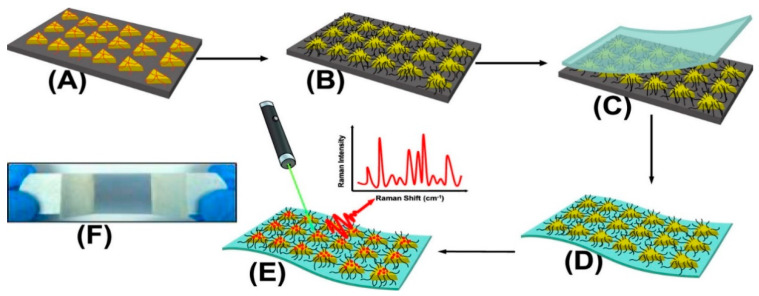
Schematic presentation of SERS substrate functionalization and drug detection process. (**A**) Chemically synthesized Au TNPs in acetonitrile are immobilized onto an APTES-functionalized glass substrate through incubation. (**B**) Au TNPs are functionalized with PEG-thiolate. (**C**) Adhesive tape is placed on the Au TNP-containing a glass substrate (**D**) Au TNPs are lift off from the glass to the tape. (**E**) human biofluids drop-casted directly onto the nanosensor resulting in physisorption of drugs onto TNPs. (**E**) SERS spectra collection. (**F**) Bluish gray area in the photograph is the plasmonic patch and the overall construct resembles with Band-Aid [94].

Masterson et al. reported a sensitive substrate for detecting cocaine, heroin, fentanyl, and their binary mixtures [94]. The substrate was prepared by first functionalizing triangular nanoprisms with poly(ethylene glycol)-thiolate in the solid-state and, subsequently, by generating flexible plasmonic patches and forming high-intensity electromagnetic hot spots (Figure 4). Sebok et al. proposed a selective substrate by adsorption of L-cysteine and L-glutathione on the surface of gold substrates for the selective detection of Ibuprofen and Dopamine, respectively [95]. Chen et al. introduced a reagent-less aptameric sensor based on SERS with “signal-on” architecture using a model target of cocaine [83]. The sensor was modified by self-assembly of 3-mercaptopropionic acid (MPA) and a 5′-terminal thiolated oligonucleotide aptamer with tetramethylrhodamine (TMR) in the presence of cocaine. Yu et al. proposed a selective substrate for quantitively monitoring the level of dopamine [96]. Citrate, as both the capping agent of Ag nanoparticles and the sensing agent of dopamine, was self-assembled on the surface of Ag dimers by reacting with carboxylic groups on the surface of Ag nanoparticles, forming a stable amide bond. A high SERS hotspot region with an intense electric field generated at the gap of the Ag nanoparticle dimers allowed for highly selective detection of dopamine. Stewart et al. modified the surface of metallic nanoparticles with different mixed thiol ratios for the selective detection of amphetamine derivatives such as MDMA [97]. The modification provided a strong covalent bond between the metallic surface and the thiol groups, resulting in a high selectivity for MDMA and a detection limit of 1.5 × 10^−5^ M. Sulk et al. used substrate functionalization for simultaneous quantification and identification of methamphetamine and amphetamine [98]. The amines functionalized with 2-mercaptonicotinic acid (2-MNA) bound to the substrate and pentachloro thiophenol (PCTP). The intensity of the Raman bands of analyte was measured relative to the Raman band of internal standard.

**Table 1:** Summary of experimental factors, studied in terms of their influence on the performance of SERS substrates.

Experimental Factor Investigated	Reference
Nanoparticle aggregation agent, aggregation time, pH	[90]
Nanoparticle size, capping agent, excitation wavelength	[49]
SERS substrate material	[49,91]
3D structure and surface topography of substrate	[51,92,94]
Chemical surface functionalization of SERS substrates	[83,93,94,95,96,97,98]

### 2.3. Drug Identification in Biological Fluids Using SERS

#### 2.3.1. Saliva

Saliva sampling is a practical approach that lends itself to noninvasive, sensitive, and in situ screening for illicit drug consumption. Oftentimes, the concentration of some common drugs of abuse is higher in saliva than in plasma. In comparison with other biofluids such as urine or blood plasma, saliva provides a faster, more straightforward, and more controllable sampling and its testing can be performed by nonmedical personnel. It could be used to detect recently ingested drugs since the average residence time of a drug in the saliva is comparable to that in blood plasma (24–48 h). There have been multiple reports regarding saliva sampling to explore illicit drugs in forensic toxicology [99,100,101]. Spectroscopy is particularly suited for the development of drug detection techniques due to its high sensitivity and ability to discriminate between drug analogues. A comparison of various spectroscopic methods in terms of their ability to detect illicit drugs in saliva samples is offered by D’Elia et al. [101]. According to that report, SERS emerges as one of the most sensitive spectroscopic techniques for drug detection in oral fluids. 

The amount of consumed cocaine can be correlated to its metabolite concentration in saliva samples. In addition, it is possible to predict the last time of cocaine use by the metabolite-to-parent drug ratio [102]. Inscore et al. described a method that could consistently detect 50 ppb of cocaine and other drugs of abuse such as diazepam, amphetamine, and phencyclidine in saliva using silver and gold doped sol-gel immobilized in glass capillaries [103]. The improvement in signal intensity was provided by electropositive silver and electronegative gold nanoparticles to alter the interaction between the drugs and the plasmonic nanostructures via attracting charged chemical groups. Farquharson et al. proposed a SERS substrate for testing 150 different drugs in saliva samples [104]. In this work, fused gold colloids trapped in a porous glass matrix contained in glass capillaries, made possible the detection of trace amounts of the target analyte. A search-and-match method was used to better screen the results, which compared the SERS spectra of the experiment to those already available. The method allowed the detection and identification of 50.0 ng/mL cocaine, 1.0 µg/mL diazepam, 10.0 µg/mL acetaminophen, and 1.0 µg/mL of phencyclidine. 

Compared to traditional SERS detection in saliva samples, integration of microfluidics with SERS results in improved signal reproducibility, allowing for the direct detection of an analyte through the interaction of the surface plasmons and the target analyte in a liquid environment.

**Figure 5:**
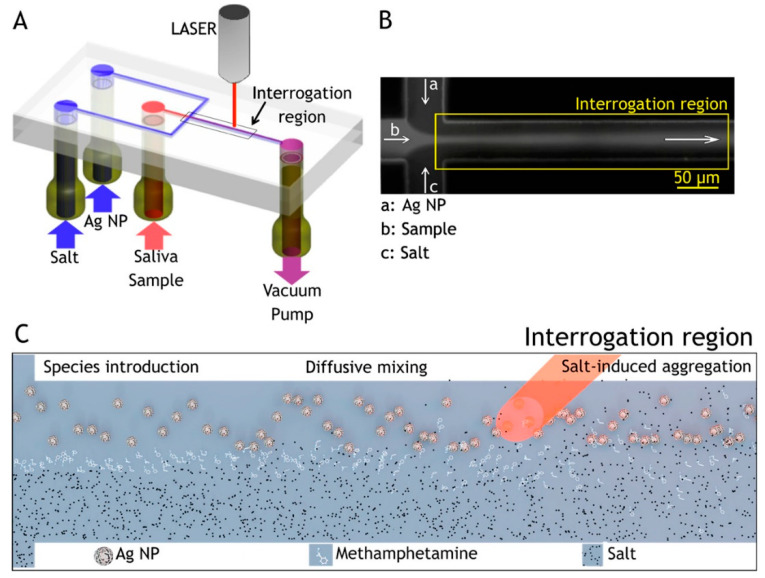
Flow-focusing microfluidic device used for controlled Ag-NP aggregation [105]. (**A**) Ag-NP suspension, a saliva sample, and salt solution are loaded in the device and driven through it by a vacuum pump. (**B**) At the flow-focusing junction, the sample stream is enveloped by the side-streams and diffusion drives lateral mass transport between the laminar flows, here visualized with a fluorescent dye. (**C**) Ag NP, analyte, and salt solution are introduced to the channel from the left and flow toward the right. Analyte molecules resident in the focused stream diffuse laterally into the side flows. Salt ions also diffuse into the colloid stream inducing controlled nanoparticle aggregation, creating SERS-active clusters that convect downstream [105].

Through integration with microfluidics, Andreou et al. developed various SERS platforms to detect drugs of abuse in saliva within minutes using Ag colloidal nanoparticles as a sensing medium (Figure 5) [105]. The device provided partial separation through analyte diffusion from the complex matrix. The concentration gradient of the chemicals, raised by laminar flow in the device, was used to control the interactions between the analyte in a saliva sample, Ag nanoparticles, and a salt. The target molecules first diffused laterally into the side flows and salts diffused into the colloid flow, allowing nanoparticles to aggregate, resulting in a sensitive detection with strong signals. In another report, D’Elia et al. proposed a solid substrate made of gold nanorods fabricated by a seed-mediated, surfactant-assisted method for identifying ultra-traces of cocaine in saliva without any sample treatments [106]. Using Orthogonal Projections to Latent Structures Discriminant Analysis (OPLS-DA) as a multivariate analysis method on samples analyzed by SERS, it was possible to categorize various cocaine concentrations without any sample preparation. The proposed device could identify cocaine at a concentration as low as 1.0 ng/mL.

The combination of SERS with solid-phase extraction (SPE) can assist in the separation of various types of illicit drugs with low concentrations in saliva [107,108]. Employing sample pretreatment methods such as physical separation, chemical separation, and SPE, Dana et al. detected concentrations of less than 25 ng/mL of cocaine in saliva. They used gold sol-gel SERS-active capillaries to fabricate SERS substrates [109]. In addition, because of the added chemicals during the experiments (chemical buffer solution amongst others), they showed that it could serve as a potential procedure to detect basic drugs and acidic drugs present in the saliva matrix.

#### 2.3.2. Urine

Urine composed of about 95% water can be used to screen for illicit drugs that entered the body 1–4 days earlier. Most synthetic drugs, including amphetamine and methamphetamine, are removed through urination [110]. Moreover, illicit drug use can also be detected by screening for the metabolites of the parent drug, which frequently remain present in urine for many hours, or even days. Despite these advantages, there are some shortcomings in analyzing urine samples for drug detection. Raman signals of uric acid, albumin, and creatinine, some of the significant urine components, can heavily interfere with the signals of low concentrations of drugs in the sample [111,112].

Because of multiple issues with urine drug testing, Riordan et al. proposed a novel method of sheath flow SERS to identify benzoylecgonine, the primary metabolite of cocaine, in urine samples [113]. This method uses hydrodynamic focusing to confine analyte molecules eluting out of a column onto a SERS planar substrate, where the molecules are detected by their unique SERS signals. Although successful in benzoylecgonine detection, the process is complex and lengthy due to the presence of more than 2000 compounds in the sample.

**Figure 6:**
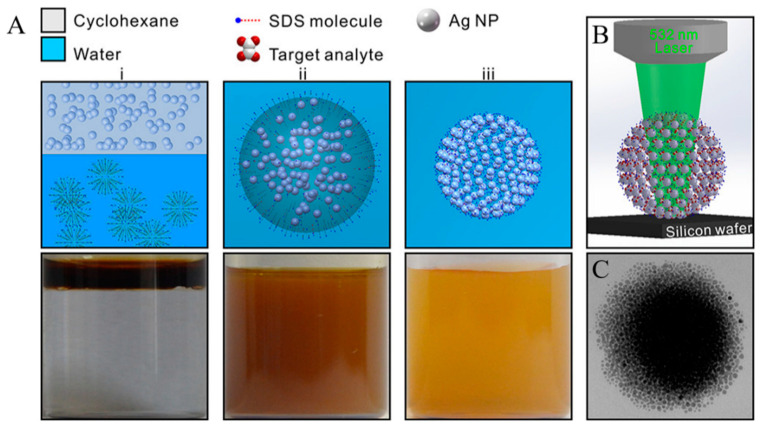
(**A**) Schematic and the corresponding optical images of the self-assembly of Ag NPs into spherical Ag colloidal superstructures. (i) The addition of CYH-dispersed Ag NPs into SDS aqueous phase. (ii) An oil-in-water emulsion through vigorous stirring. (iii) The as-prepared sols of spherical superstructures after the evaporation of oil. (**B**) Schematic of SERS platform for sensing analytes located in the 3D geometrical gaps of colloidal superstructures. (**C**) TEM image of a single 3D colloidal superstructure [116].

Portable Raman spectrometers are gaining ground rapidly in forensic analysis applications. Although less efficient than their bench-top counterparts housed in the laboratory, they are easier to use by law enforcement personnel and health professionals. An overview of different modes of Raman spectroscopy, including spatially offset Raman spectroscopy (SORS), Resonance enhanced Raman spectroscopy (RERS), SERS, and their in-the-field applications in the homeland security and detection of chemical and biological hazards can be found in [114]. Miniaturized Raman systems capable of performing in situ analysis of forensic, pharmaceutical and art samples have been around for over ten years [115]. More recently, Han et al. proposed a portable kit for on-site detection of amphetamine in human urine [116]. The package included a sample-preparation platform to extract the analyte from urine by cyclohexane (CYH) and a transportable Raman device (Figure 6). Simultaneously, spherical colloidal superstructures were formed by assembling monodispersed Ag nanoparticles in the CYH aqueous phase creating SERS hotspots between every two adjacent particles in 3D space. An enhancement factor greater than 10^7^ combined with high enrichment of drug molecules in 3D hotspots, excellent stability, and high reproducibility turned the device into a suitable SERS platform for quantitative analysis of amphetamine in both human urine and aqueous solutions. Amphetamine was detected with a detection limit as low as 10 ppb, corroborated by UPLC (Ultra Performance Liquid Chromatography) assays.

In work conducted by Dong et al., the advantages of sample preparation and portable systems were combined with the SVM classification method for the trace detection of MDMA and methamphetamine in human urine samples [117]. Urine samples containing methamphetamine and MDMA were mixed with gold nanorods (GNRs) stabilized with polyethylene glycol methyl ether thiol (PEG-SH). GNRs caused a considerable enhancement in SERS signals using a D-SERS platform. SVM enabled identification in complex matrices without sample pretreatment. The model identified the target analytes in the urine of drug users with an accuracy higher than 90%. The importance of D-SERS for the detection of illicit drugs has been highlighted elsewhere [52,118]. Mostowtt et al. demonstrated a SERS platform for identifying four synthetic cannabinoids with relatively similar structures in human urine and aqueous solution samples [48]. Mixing the analytes with gold nanoparticles prepared in alkaline or alkali earth salt solutions resulted in the nanoparticles’ aggregation and formation of spectral hotspots. The method resulted in distinct SERS spectra for each of the cannabinoids with the limit of the detection of as low as 18 ng/mL. Alharbi et al. developed a SERS substrate to detect tramadol, a narcotic painkiller, in a urine sample [54]. Aggregating agents, aggregation times, incubation times, and pH were optimized step by step to define the best parameters. Finally, hydroxylamine silver nanoparticles, 0.5 M NaCl as an aggregating agent, and neutral pH were chosen as the optimum parameters. The limits of detection for tramadol in water and artificial urine were calculated to be 5 × 10^−4^ M and 2.5 × 10^−6^ M, respectively. 

A combination of liquid-liquid chromatography with SERS was also used for identifying drugs in urine samples [44,119]. Cocaine, heroin, amphetamine and pharmaceuticals such as procaine and (nor-) papaverine extracted with HPLC were detected in quantities down to 1 μg with SERS performed in the wells of microtiter plates containing the analyte and a gelatin matrix-stabilized silver halide dispersion [120]. The same research group also showed that the combination of HPLC extraction and SERS-based detection can be used for the characterization of small quantities (1 μg/domain) of several drugs (Carbamazepine, Methadone, etc.) and some of their degradation products found in blood and urine [44].

#### 2.3.3. Blood

Contrary to urine and saliva samples, detecting and quantifying illicit drugs in human blood is complex and challenging. Blood plasma produces strong SERS spectra that interfere with drug signals, requiring rigorous sample extraction procedures [121].

**Figure 7:**
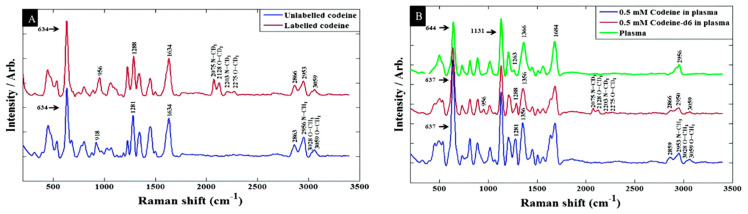
Baseline-corrected SERS spectra of 100 μM codeine spiked into (**A**) water (**B**) human plasma [122].

Trachta et al. took advantage of the combination of HPLC as the separation technique and SERS to analyze drugs in human blood samples from silver halide dispersions deposited in the wells of microtiter plates [119]. A gradient technique based on a methanol/buffer mixture was developed to lower the limit of detection of the investigated drugs into the 1 µg/sample domain. Using HPLC to extract drugs from the blood serum of patients, Zhao et al. also showed that quantities as small as a few hundred nanograms can be detected for eight different analytes of the benzodiazepine family by using “gold films over nanospheres” (AuFONs) SERS-active substrates with an FT-NIR (1064 nm wavelength) Raman spectrometer [120]. Subaihi et al. employed SERS combined with multivariate statistical analysis to detect and quantification of ß-blocker propranolol in human plasma samples [1]. Followed by PCA and PC-DFA, the SERS spectra clearly distinguished propranolol in a concentration range of 0 to 120 µM, spiked into human plasma. The limit of detection for the propranolol was 0.53 μM. In more recent work, they added a definite quantity of isotopically labeled codeine as an internal standard to enhance the accuracy of the detection of codeine in blood plasma [122]. A silver colloidal system with sodium chloride as the aggregation agent was used for SERS enhancement. Particularly, partial least squares regression (PLSR), as a multivariate statistical approach, was used to analyze data. The limit of detection of codeine in plasma and water were 416.12 ng/mL and 209.55 ng/mL, respectively. The results are shown in Figure 7.

Table 2 summarizes the methods reported above, along with their detection and performance characteristics.

## 3. Summary and Outlook

Over the past couple of decades, SERS has emerged as a promising analytical tool for clinical and forensic applications. The technique combines the advantages of high sensitivity with fluorescence background quenching, thus overcoming many of the shortcomings of conventional Raman spectroscopy. SERS is a mode of vibrational spectroscopy that offers the sensitivity required for detecting and quantifying trace levels of illicit drugs in biological fluids or aqueous samples. Moreover, it lends itself to applications that require rapid, in situ, non-destructive, and accurate detection of target compounds in various samples. The ability to implement SERS by employing a variety of nanoparticles and substrates that can be created in many ways also adds to the method’s versatility.

Despite all the advancements, challenges still exist regarding the application of SERS in routine forensic analyses. Uniform and reproducible SERS signals depend highly on the optimization and stabilization of the substrates. Colloidal substrates lack reproducibility but have high enhancement factors for SERS signals. Nowadays, there is more control over the shape of the nanoparticles and hotspots, making the creation of reproducible substrates possible. The proximity of the nanoparticles to the plasmonic surface and surface coverage are other issues that must be addressed to enhance SERS detection performance. Moreover, drug samples often exist in small quantities and rarely as pure compounds. Since the adsorption of molecules on the surface is highly competitive, there must be effective strategies such as functionalization of the substrate to selectively capture the target analyte on the surface. 

SERS also has certain limitations that may reduce its sensitivity. Most biological samples exhibit strong fluorescence in the visible light region, which lowers sensitivity. Moreover, target molecules in complex matrices, such as biological fluids, are often masked by the presence of other components in the sample that prevent their accurate characterization through vibrational spectroscopy [121,123]. One way to overcome such obstacles is the integration of SERS with separation techniques, such as thin-layer chromatography (TLC) [124,125], HPLC [119], chemical separation [98], and solid/liquid-phase extraction [126]. Another way is to use capture methods for selective detection and recognition of the target molecules combined with SERS. Common capturing techniques for illicit drug detection are molecular imprinting [127] and employing aptamer [128,129] and antibodies [128]. Other techniques, such as the incorporation of microfluidics [130,131] for enhancing the interaction between the analyte and SERS substrate and colorimetric assays [132] as a prescreening step, have been employed to enhance SERS signals. When used together with other analytical methods, such as fluorescence spectroscopy and colorimetry, SERS can significantly improve the sensitivity and discriminatory power of chemical analysis [133,134,135]. Finally, integration of SERS with powerful analytical machine learning techniques helps extract relevant, fast, and more accurate results for on-site drug detection, thus popularizing its use even among non-expert users. Such techniques include artificial neural networks (ANNs) [136], support vector machines (SVM) [53,54], partial least squares (PLS) [137], principal component analysis (PCA) [138], and principal component-discriminant function analysis (PC-DFA) [134]. Moreover, the combination of SERS with chemometric algorithms facilitates quantification analysis by extracting and comprehending complex SERS fingerprints [139,140].

The increasing rate of illicit drug use, its devastating consequences for the health of people who use drugs, and its broader risk to the well-being of our societies create the urgent need to adopt sensitive yet simpler, analytical drug detection methods. The purpose of this article was to summarize the contribution of SERS-based strategies on that front by reviewing the progress made to date towards the detection of drugs of abuse in various samples, including biological fluids, such as urine, blood, and saliva. An overview of the SERS-active substrates employed to date for demonstrating drug detection has also been presented. Recent work in the field has established the great potential of SERS to serve not only as a standard laboratory method but also as a mobile platform for drug detection, owing to recent advances in the performance of handheld Raman spectrometers. Similar to many other chemical analysis methods, SERS is also not devoid of shortcomings, and there are still unresolved challenges regarding its widespread application. Current efforts to integrate SERS with chemically functionalized substrates and statistical analysis methods are a step in the right direction and are expected to dramatically improve the selectivity and discriminatory ability of this spectroscopic technique. 

## Figures and Tables

**Table 2:** Applications of SERS analyses of illicit drug detection.

Drug	Matrix	Analysis Type	SERS Substrate	Laser Line (nm)	Limit of Detection	Reference
Amphetamine	Aqueous solution	Quantitative	Ag colloidal solution	532	5 µg	[23]
Benzocaine	Aqueous solution	Quantitative	Au@Ag nanocube-based plasmene nanosheets	514	0.9 × 10^−6^ gr·cm^−2^	[91]
Cannabinol	Aqueous solution	Quantitative	vertically aligned hexagonally close-packed AuNR arrays	632.8	1 µM	[86]
Cannabinoids	Aqueous solution	Quantitative	Colloidal AuNPs	785	18–60 ng·mL^−1^	[74]
Chrysoidin	Aqueous solution	Quantitative	AuNSt-GO-AuNSt sandwich structure	785	1 nm	[120]
Cocaine	Saliva	Semi- quantitative	Au doped sol-gel capillary	785	50 ppb	[80]
Cocaine	Human saliva	Semi- quantitative	fused gold colloids trapped in a porous glass matrix	785	50 ng·mL^−1^	[75]
Cocaine	Saliva	Quantitative	gold nanorods colloidal solution	780	10 ng·mL^−1^	[54]
Cocaine	Aqueous solution	Quantitative	(AuNP)-embedded paper swab	785	0.6 ng	[23]
Cocaine	Saliva	Quantitative	Dendritic silver nanostructures	632.8	100 ppb	[90]
Cocaine	Human Urine	Semi- quantitative	Self-assembly of 2D AuNPs film	633 nm	500 ppb	[121]
Cocaine	Aqueous solution	Semi quantitative	Colloidal AuNPs integrated with microfluidic device	633	4.6 ng·mL^−1^	[122]
Cocaine	Aqueous solution	Quantitative	Ag colloidal solution	532	5.0 µg	[122]
Codeine	Human Saliva	Quantitative	Au doped sol-gel capillary	785	25 ng·mL^−1^	[109]
Codeine	Human Plasma	Quantitative	Colloidal AgNPs	633	1.39 µM	[123]
Dopamine	Aqueous solution	Quantitative	Colloidal ANPs	532	20 pM	[113]
Erythrosine B	Aqueous solution	Quantitative	AuNSt-GO-AuNSt sandwich structure	785	1 nm	[121]
Fentanyl	Aqueous solution	Quantitative	(AuNP)- embedded paper swab	785	1.0 ng	[80]
Fentanyl	Aqueous solution	Quantitative	Dendritic silver nanostructures	632.8	0.078 ppm	[103]
Fentanyl	Urine	Quantitative	AuNPs assembled on filter paper	785	10 ppb	[92]
MDMA	Aqueous solution	Quantitative	D-SERS		10 µM	[76]
MDMA	Human Urine	Quantitative	2D-GNR assembled by (mPEG-SH) capping	785	0.1 ppm	[45]
MDMA	Aqueous solution	Quantitative	Colloidal AgNPs modified by thiols	785	1.5 × 10^−5^ M	[89]
MDMA	Human Urine	Semi-quantitative	Au nanorods stabilized with SH-PEG	785	0.1 ppm	[55]
Meperidine	Aqueous solution	Quantitative	Ag colloidal solution	532	3 µM	[23]
Methadone	Human plasma	Semi-quantitative	Silver halide dispersed into the wells of microtiter plates	-	1 µg/sample	[44]
Methamphetamine/2-MNA	Aqueous solution	Quantitative	Etched Ag foil	633 nm	17 ppm	[45]
Methamphetamine	Human Urine	Semi-quantitative	Au nanorods stabilized with SH-PEG	785	0.1 ppm	[55]
Methamphetamine	Human saliva	Semi-quantitative	Colloidal AgNPs integrated with microfluidics	633	10 nm	[106]
Morphine	Aqueous solution	Semi-quantitative	Colloidal AuNPs integrated with microfluidic device	633	13 ng·mL^−1^	[75]
Tramadol	Artificial Urine	Quantitative	Hydroxylamine AgNPs	633 nm	2.5 × 10^−6^ M	[78]
Tramadol	Aqueous solution	Quantitative	Hydroxylamine AgNPs	633 nm	5 × 10^−4^ M	[78]
Phencyclidine	Human saliva	Sem-quantitative	fused gold colloids trapped in a porous glass matrix	785	1 µg·mL^−1^	[106]
